# Left hand extensor tenosynovitis due to *Histoplasma capsulatum* complicated by immune reconstitution inflammatory syndrome

**DOI:** 10.5194/jbji-6-355-2021

**Published:** 2021-09-23

**Authors:** Talha Riaz, Mark Collins, Mark Enzler, Marco Rizzo, Audrey N. Schuetz, Julia S. Lehman, Douglas Osmon, Irene G. Sia

**Affiliations:** 1 Division of Infectious Diseases, Mayo Clinic, Rochester, Minnesota, USA; 2 Divisin of Infectious Diseases, University of Arizona Medical Center, Tucson, AZ, USA; 3 Department of Radiology, Mayo Clinic, Rochester, Minnesota, USA; 4 Division of Hand Surgery, Mayo Clinic, Rochester, Minnesota, USA; 5 Department of Laboratory Medicine and Pathology, Mayo Clinic, Rochester, Minnesota, USA; 6 Department of Dermatology, Mayo Clinic, Rochester, Minnesota, USA

## Abstract

We describe a case of left hand extensor tenosynovitis due to histoplasmosis in a patient with dermatomyositis on chronic immunosuppression. Treatment involved surgical debridement and antifungal therapy. The patient experienced paradoxical worsening of tenosynovial inflammation during de-augmentation of immunosuppression felt to be immune reconstitution inflammatory syndrome.

## 1 Brief history of the present illness

A 46-year-old woman with dermatomyositis diagnosed 5 months earlier, presented with a 1-month history of pain, warmth, redness and swelling localized to the metacarpophalangeal (MCP) joints and proximal interphalangeal (PIP) joints along with limitation of the movement of fingers and wrist. There was intermittent low-grade fever, occasional night sweats and recent onset of dry cough and loose stools over the past 1 week. Medications were prednisone 60 mg per day and mycophenolate mofetil (MMF) 1500 mg twice daily for the past 4 months, with hydroxychloroquine 200 mg twice daily added 2 weeks prior due to worsening symptoms of dermatomyositis including fatigue, as well as trimethoprim/sulfamethoxazole (TMP/SMX) 800/160 mg thrice weekly for *Pneumocystis jirovecii* prophylaxis.

Patient was born and raised in the upper midwestern United States, married with four healthy children and worked as a corporate meeting planner. One year prior to presentation, the patient went camping and swam in a freshwater lake. There was occasional gardening prior to the diagnosis of dermatomyositis. There was no exposure to fish tanks. Travel was extensive throughout the US including Iowa, Illinois, California, Nevada, Arizona, Texas and Georgia.

**Figure 1:**
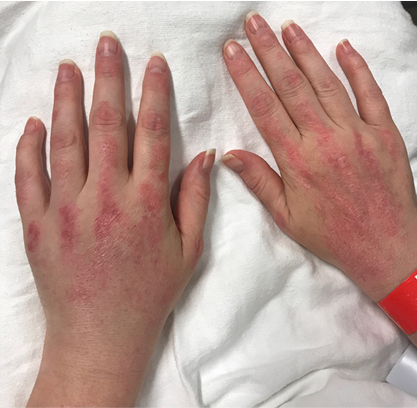
Macular violaceous erythema over the bilateral extensor hands in a patient with dermatomyositis on the dorsal surfaces of both hands.

On physical examination, the patient appeared well and was not in acute distress. Blood pressure was 134/80 mm Hg, heart rate 77 beats per min, respiratory rate 18 breaths per minute and temperature 36.9 ${}^{\circ }$C. Examination of the left upper extremity noted swelling about the ulnar aspect of the left dorsal hand. There was pain with gentle range of motion about the ulnar aspect of left wrist and fourth and fifth MCP joints; no pain was elicited with gentle passive range of motion about any of the other joints of her left hand. There was no active synovitis in other joints. Sensation to light touch was intact in the median, ulnar and radial nerve distributions. A rash consistent with dermatomyositis was present on the chest, shoulders, forearm and dorsal aspect of both hands over the MCP joints (see Fig. 1). No open wounds were seen. All other systems were within normal limits.

The white blood cell count was $7.6\times 10^{9}/\;L$ (range $3.4\times 10^{9}$ to $9.6\times 10^{9}/\;L$), and platelet count was $292\times 10^{9}/\;L$ (range $157\times 10^{9}$ to $371\times 10^{9}/\;L$). Sedimentation rate was 32 mm/h (range 0–29 mm/h), and c-reactive protein (CRP) was 52 mg/L (reference range ≤ 8 mg/L). Alanine aminotransferase was 77 U/L (range 7–45 U/L), aspartate aminotransferase was 28 U/L (range 28–43 U/L) and alkaline phosphatase was 51 U/L (range 37–98 U/L). QuantiFERON-TB Gold Plus was indeterminate, and human immunodeficiency virus (HIV) screen was negative. An X-ray of the chest was clear.

**Figure 2:**
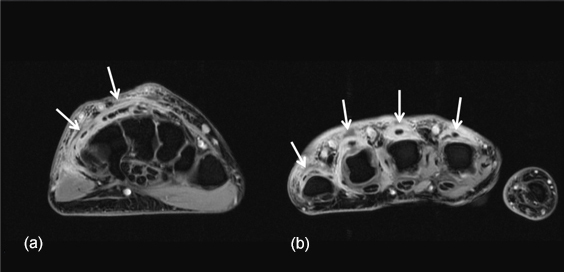
Axial post-contrast T1-weighted fat-suppressed images at the level of the wrist **(a)** and metacarpal heads **(b)** demonstrate irregular thickened tenosynovial enhancement involving the extensor tendons of the second to fifth fingers (white arrows). The flexor tendons are normal by comparison.

An ultrasound of the left hand was notable for tenosynovitis of the left fourth dorsal compartment and subcutaneous edema of her dorsal left hand. On post-contrast T1-weighted fat-suppressed magnetic resonance imaging (MRI) of the left hand, there was peripherally enhancing tenosynovitis involving the extensor compartment (Fig. 2). There was no evidence of underlying septic arthritis or osteomyelitis.

**Figure 3:**
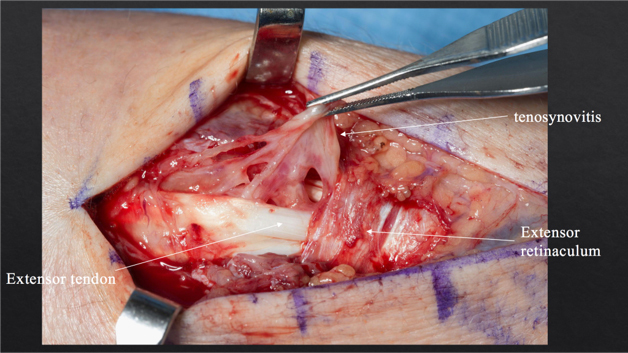
Intraoperative photograph showing inflammation in the fourth extensor compartment and tenosynovitis.

Orthopedic hand surgery was consulted. The patient underwent tenosynovectomy of the extensor tendons of the left wrist and hand along with irrigation and debridement. Upon exposing the extensor mechanism of the left hand, inflammation was noted in the fourth extensor compartment with edema and fluid but no frank pus (Fig. 3). Multiple specimens were sent for bacterial, fungal and mycobacterial cultures as well as Gram, fungal and acid-fast stains on fresh tissue.

Surgical pathology from the tenosynovium revealed fibrinous exudate and severe chronic inflammation. Gram, fungal and mycobacterial stains on fresh tissue were negative.

There was no growth on bacterial cultures. Fungal cultures revealed the growth of *Histoplasma capsulatum* at day six. Serology for *Blastomyces, Aspergillus* and *Sporothrix* were negative; *Histoplasma* serology and urine antigen were positive (Table 1). The patient was started on itraconazole 200 mg twice daily and was gradually being weaned off prednisone.

**Figure 4:**
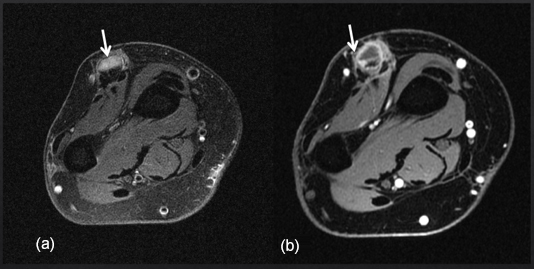
Axial T2-weighted fat-suppressed image **(a)** and corresponding post-contrast T1-weighted fat-suppressed image at the level of the distal forearm **(b)** obtained 12 months later after repeat surgical debridement procedures and antifungal therapy demonstrate an intermediate T2-signalling, hypoenhancing inflammatory nodule (white arrows) overlying the common extensor tendons.

There was a good initial response to antifungal therapy and itraconazole levels were within therapeutic range. Three months following the initial left hand debridement, the patient presented with a new nodule and a 1.2 cm fluid collection on the third MCP joint of the left hand; MRI was suggestive of worsening tenosynovitis (Fig. 4).

Patient underwent additional irrigation and debridement; there was a thick rind of tissue around the extensor tendons of fourth and fifth compartments. Fungal smear of a fresh sample from the infected fluid collection showed many yeast forms; all of the fungal cultures remained negative. Surgical pathology was notable for necrotizing granulomatous inflammation but no fungal forms were noted on the surgical pathology specimens at that time. Itraconazole was continued. At 6 months after the initial debridement, the patient was weaned off steroids and mycophenolate and was started on monthly immunoglobulin therapy for dermatomyositis.

**Figure 5:**
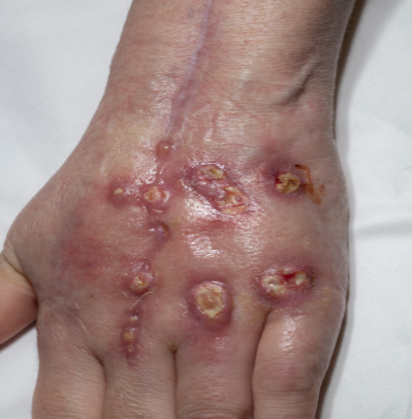
Multiple ulcerative violaceous edematous nodules on the dorsal hand in a patient with dermatomyositis and histoplasmosis-associated extensor tenosynovitis.

Soon after completely discontinuing steroids at 6 months of antifungal therapy, the patient presented with weeping, nodular, white lesions along the surgical incision marks (Fig. 5). Urine *Histoplasma* antigen as well as serologies showed improvement (Table 1). Nevertheless, there was concern about possible clinical progression of the infection while on itraconazole.

**Figure 6:**
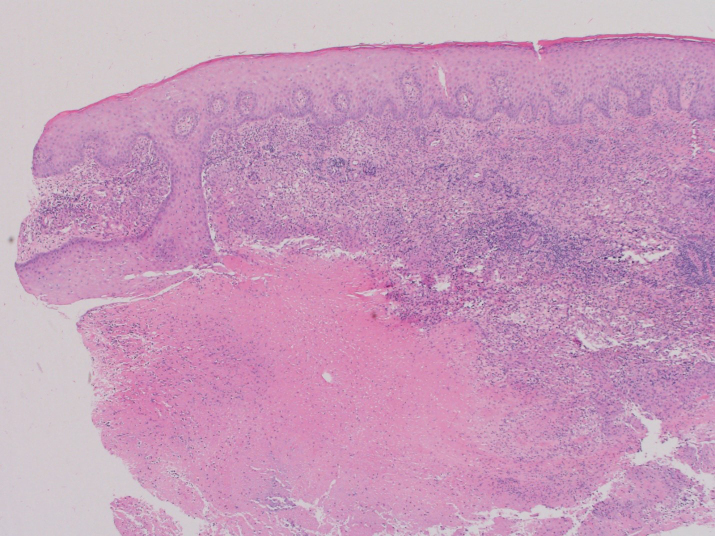
Hematoxylin and eosin (H&E) stain of skin punch biopsy demonstrates necrotizing granulomatous inflammation (40× magnification).

**Figure 7:**
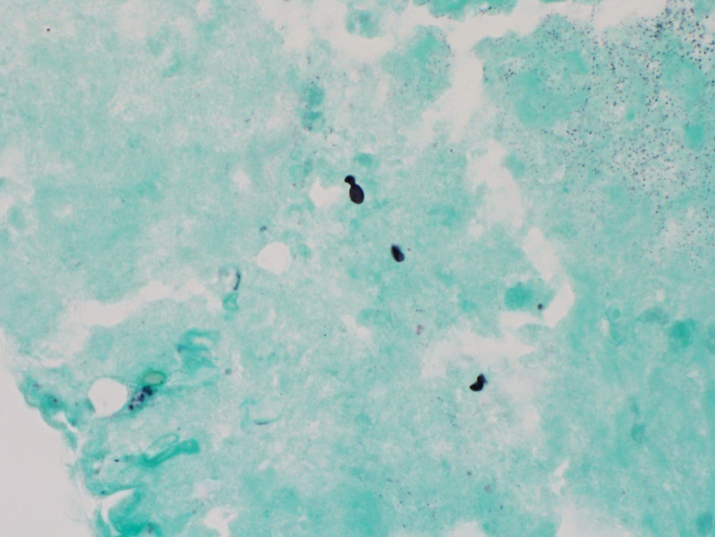
GMS stain of skin punch biopsy highlights small, oval yeast forms, some with budding, consistent with *Histoplasma* spp. (1000× magnification).

Dermatologic punch biopsy of one of these lesions revealed necrotizing granulomatous inflammation (Fig. 6) with small, budding yeasts seen on Grocott's methenamine silver (GMS) stain, consistent with *Histoplasma* (Fig. 7). An indium-labeled white blood cell scan did not reveal focal bone uptake in the left hand, although there was significant soft tissue uptake. The patient underwent repeat tenosynovial debridement and intraoperatively was found to have extensive inflammation and erosions of the fourth extensor compartment tendons in the dorsum of the left hand. Postoperatively, the patient was started on intravenous liposomal amphotericin B overlapped with oral delayed release posaconazole 300 mg per day. Treatment was complicated by severe electrolyte abnormalities, which prompted discontinuation of liposomal amphotericin B. Fungal cultures were negative yet again. Serum and urine *Histoplasma* antigen revealed stable low-level positives (see Table 1, at 6 months). There was a concern that the fistulizing tracks with tenosynovitis could represent immune reconstitution inflammatory syndrome (IRIS). In view of this, she was administered oral prednisone for suspected IRIS with a plan for a prolonged taper. TMP/SMX for *Pneumocystis* prophylaxis was re-started as well. After 4 weeks, posaconazole was changed back to itraconazole 200 mg twice per day due to cost issues.

**Figure 8:**
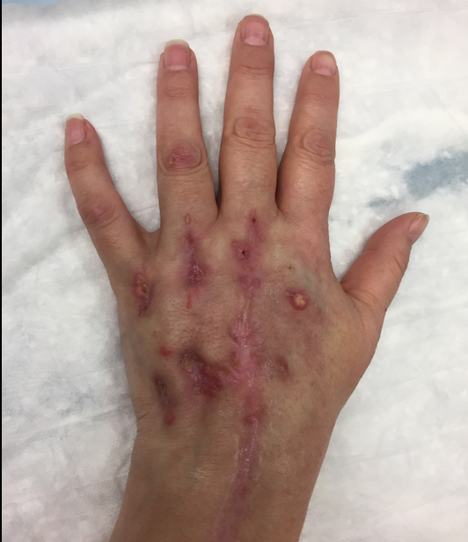
Residual violaceous indurated scars on the dorsal hand in regions of previous suppurative and ulcerative lesions of histoplasmosis-associated extensor tenosynovitis in a patient with dermatomyositis. A single suppurative lesion remained on the medial dorsal hand.

At the time of the patient's last follow-up, the suppurative and ulcerative lesions on the left dorsal hand, as well as the edema, had mainly resolved, leaving indurated violaceous scars (Fig. 8). A plan for a prolonged steroid taper was proposed in the setting of probable IRIS. The anticipated duration of therapy with itraconazole was 1 year assuming that the elevated serum and urine *Histoplasma* antigen levels completely resolved.

**Table 1:** Trend in *Histoplasma* serology and urine antigen. CF: complement fixation.

Time	*Histoplasma* CF	*Histoplasma* CF	*Histoplasma*	Urine *Histoplasma* antigen (ng/dL)
	mycelial antibody titer	yeast antibody titer	immunodiffusion	reference ≥ 0.50 = positive; 0.00–0.10 = negative
0 month	1: 1024	1: 512	H and M band +	2.04
3 months	1: 512	1: 512	H and M bands +	1.26
6 months	1: 246	1: 256	H and M bands +	0.05
9 months	1: 256	1: 256	H and M bands +	0.08

## 2 Discussion

*Histoplasma capsulatum* is a dimorphic fungus that is endemic in the Ohio and Mississippi river valleys. It is primarily an intracellular pathogen infecting macrophages and causes granulomatous inflammation. Transmission typically occurs via inhalation of spores. Following invasion of the lungs, it can then disseminate hematogenously to extrapulmonary sites. However, the majority of human infections are asymptomatic, except in patients with altered immunity, such as individuals with acquired immunodeficiency syndrome (AIDS), hematologic malignancies, transplant recipients and patients receiving prolonged steroids and tumor necrosis factor (TNF) alpha blocker therapy, who are at risk for progressive infection (Wheat et al., 1982; Wheat, 1997; Assi et al., 2007; Vergidis et al., 2015).

As seen in our patient, isolated extrapulmonary organ involvement with wrist tenosynovitis due to histoplasmosis, though uncommon, has been described in literature (Cucurull et al., 2005; Vitale et al., 2015; O'Shaughnessy et al., 2017). Our patient reported an episode of respiratory tract illness 1 year prior to presentation that may have represented acute pulmonary histoplasmosis. Patient was also from a *Histoplasma*-endemic region of the US. High dose steroids and MMF for the treatment of dermatomyositis probably led to re-activation of *Histoplasma* infection that might have survived intracellularly in the macrophages. When considering fungal tenosynovitis, it is important to obtain a thorough travel and exposure history since *Histoplasma* is endemic in the midwestern U.S. A table summarizing published cases of *Histoplasma* tenosynovitis among non-HIV immunosuppressed patients is included (Table 2).

Diagnosis of histoplasmosis requires tissue for fungal culture (gold standard) and fungal smear where yeast forms (2–4 µm) may be seen with narrow-based budding. Since fungal culture may require 4 to 6 weeks for growth, a positive smear may provide early evidence of infection. Pathology generally shows granulomatous inflammation with necrotizing granulomas. Testing for *Histoplasma* antigen in urine and serum should also be performed as the presence of *Histoplasma* antigen in the urine or plasma is suggestive of disseminated disease. Serologic testing including complement fixation and immunodiffusion may further support a histoplasmosis diagnosis. Assessment for suspected wrist tenosynovitis may include ultrasound to assess for fluid collection and MRI which would typically show enhancement on T2-weighted images around the inflamed tendons. MRI is also helpful to look for bony changes consistent with osteomyelitis.

**Table 2:** Cases of Histoplasma tenosynovitis among non-HIV patients on immunosuppression.

Cases in	Age, sex	Risk	Key	Symptoms	Surgical	Antifungal	Complication	Follow-up	PMID
Literature		factor	features	duration tilldiagnosis	treatment	drug			(reference)
Case 1	42, F	Crohn's disease, on Adalimumab and Azathioprine	Left hand swelling and pain, MRI noted for flexortenosynovitis	3 weeks	Urgenttenosynovectomy	Itraconazole	–	Doing well at 1-month follow-up	32498777 (Rieth et al., 2020)
Case 2	42, F	SLE${}^{a}$ (off immunosuppression) for 2 years, takes hydroxychloroquine (SLE${}^{a}$ by itself a potential risk factor)	Left hand swelling and pain, MRI noted for flexor tenosynovitis	3 months	None (underwent biopsy)	Itraconazole 200 mg × 12 months	–	Recovered	15696561 (Cucurull et al., 2005)
Case 3	50, F	Takayasu's arteritis and ulcerative colitis, oninfliximab, methotrexate and low-dose prednisone	Right palm/wrist swelling and pain	6 weeks	Tenosynovectomy ofall flexor tendons/palm and wrist	Itraconazole	–	–	29419449 (Woods et al., 2018)
Case 4	32, F	SLE${}^{a}$ on MMF${}^{b}$ and prednisone 30 mg/d	Right forearmswelling/pain andredness, later left wrist swelling/pain	2 months	R carpal tunnel release, lost to follow-up,later L carpal tunnel release/tenolysis	Itraconazole 200 mgtwice a day for12 months	3 months after firstsurgery, presented with left wrist, ankle and knee pain, thoracicspine lesions (PDH${}^{c}$)	Doing well at 2 months follow-up (after second surgery)	23065146 (Lim et al., 2013)
Case 5	48, F	Sjogren's disease, ILD${}^{d}$ on MMF${}^{b}$ and recent steroids	Right hand/wrist swelling, pain	5.5 months	Carpal tunnel release/tenosynovectomy	IV liposomalamphotericin B, later, itraconazole for 1 year	Recovered	Doing well at 1 yearfollow-up	25762883 (Vitale et al., 2015)
Case 6	43, F	Rheumatoid arthritis, scleroderma,glucocorticoid therapy, and malignant lymphoma/chemotherapy	Right anterior tibialis and lateral peroneal tendon swelling	–	None (had a biopsy)	Amphotericin × 6 months, no effect, switched toitraconazole for12 months	Tendons normal at1 year of itraconazoletherapy	Recovered	16807041 (Filali et al., 2006)
Case 7	51, M	SLE, on MTX${}^{e}$, HDQ${}^{f}$ and rituximab	Swelling, firmness anderythema right distal arm	6 months	Fascial synovectomy	Itraconazole 200 mg twice a day for 1 month and 600 mg daily for second month	Wound non-healing 1 month after surgery, dose of itraconazole increased to 600 mg	–	32202145 (Huayllani et al., 2021)
Case 8	71 M	Chronic prednisone (5–15 mg) for emphysema	Right wrist tenosynovitis	12 months	Synovectomy	Initially itraconazole, later amphotericin B (total dose) 1140 mg	Chronic osteomyelitis of the scaphoid, requiring prox. carpectomy	No recurrence at 2 years	10515656 (Houtman et al., 1999)

Abbreviations: ${}^{a}$ Systemic lupus erythematosus, ${}^{b}$ Mycophenolate Mofetil, ${}^{c}$ Progressive disseminated histoplasmosis, d Interstitial lung disease, ${}^{e}$ Methotrexate, ${}^{f}$ Hydroxychloroquine

Treatment of tenosynovitis due to *H. capsulatum* requires a combination of surgical debridement along with antifungal therapy. Intraoperative findings include inflamed and fibrinous synovium. Initial antifungal therapy for severe disease is with lipid-complexed amphotericin for at least 2 weeks which is transitioned to oral itraconazole therapy. For mild to moderate disease, it may be reasonable to use itraconazole as monotherapy. Duration of antifungal therapy is determined by clinical response and typically is at least 12 months. In a review of 98 cases of histoplasmosis in the context of TNF alpha blocker therapy, at least 12 month of antifungal therapy was suggested (Vergidis et al., 2015). Along with the antifungal therapy, de-augmentation of immunosuppression is recommended for as long as is feasible. It is reasonable to resume immunosuppression following diagnosis of histoplasmosis after the patient has received at least 1 year of antifungal therapy and has achieved undetectable antigen levels.

The cutaneous IRIS manifestations while being weaned off of immunosuppression can be compared to patients with HIV who developed disseminated cutaneous histoplasmosis following initiation of anti-retroviral therapy (Passos et al., 2011; Kiggundu et al., 2016). In a recent study from French Guiana, 22 cases of histoplasmosis-related IRIS were described (Melzani et al., 2020). IRIS has been described in 9 out of 98 (9.2 %) non-HIV infected patients with histoplasmosis, with a median time to onset of IRIS after TNF alpha blockade of 6 weeks (Vergidis et al., 2015). Another report described 19 cases of histoplasmosis-complicating TNF alpha therapy of whom 8 (42 %) developed IRIS (Hage et al., 2010). It is the reduction in immunosuppression that brings the onset of IRIS. This paradoxical worsening can be misinterpreted as treatment failure, as was seen in our patient who was started on amphotericin B (complicated by electrolyte abnormalities) following surgical re-debridement before eventually being determined to have IRIS. Our treatment approach included re-initiation of oral steroid therapy to manage the IRIS along with continuation of antifungal therapy.

In summary, upper extremity swelling, pain and warmth in immunocompromised patients warrants special attention. Although uncommon, *Histoplasma* tenosynovitis warrants a high index of suspicion, especially in patients from endemic regions. It is typically treated with open debridement and a prolonged course of antifungal therapy along with discontinuation of immunosuppression. Relapse rate is 30 % according to one study (O'Shaughnessy et al., 2017). Therefore, close posttreatment follow-up is needed.

## Data Availability

No data sets were used in this article.
